# Closing the carbon cycle through rational use of carbon-based fuels

**DOI:** 10.1007/s13280-015-0728-7

**Published:** 2015-12-14

**Authors:** J. M. Don MacElroy

**Affiliations:** UCD School of Chemical and Bioprocess Engineering, University College Dublin, Belfield, Dublin 4, Ireland

**Keywords:** Carbon capture and recycle, Carbon dioxide utilization, Methane hydrates, Natural gas, Shale gas

## Abstract

In this paper, a brief overview is presented of natural gas as a fuel resource with subsequent carbon capture and re-use as a means to facilitate reduction and eventual elimination of man-made carbon emissions. A particular focus is shale gas and, to a lesser extent, methane hydrates, with the former believed to provide the most reasonable alternative as a transitional fuel toward a low-carbon future. An emphasis is placed on the gradual elimination of fossil resource usage as a fuel over the coming 35 to 85 years and its eventual replacement with renewable resources and nuclear power. Furthermore, it is proposed that synthesis of chemical feedstocks from recycled carbon dioxide and hydrogen-rich materials should be undertaken for specific applications in the transport sector which require access to high energy density fuels. To achieve the latter, carbon dioxide capture is imperative and possible synthetic routes for chemical feedstock production are briefly reviewed.

## Introduction

In the current inventory of energy usage across the globe, fossil resources account for over 80 % of our requirements. Furthermore, it is envisaged by many that by 2050 the world’s energy needs will still be served in large part by fossil fuels; for example, the International Energy Agency’s Energy Technologies Perspectives^1^ suggests that by 2050, 50 % of the total energy supply will still be from fossil fuels. Notwithstanding these projections, a number of reviews, including the recent Intergovernmental Panel on Climate Change (IPCC 2014) report, have clearly stated that as a whole we are failing to direct our consumption along a sustainable energy path. It is becoming abundantly clear that if we are to curtail carbon emissions, while maintaining and/or encouraging economic growth both in the developed world and in the less developed economies, a balance must be struck between the use of fossil sources, continued use, and further development of nuclear power generation and the implementation of renewable energy technologies. The focus of this article will be on how we might technically reduce CO_2_ emissions (or indeed close the anthropogenic carbon cycle) over the next four decades and beyond while still making use (at a gradually decreasing level) of fossil reserves. There are two strategies which could be adopted to achieve this target.

**Fig. 1:**
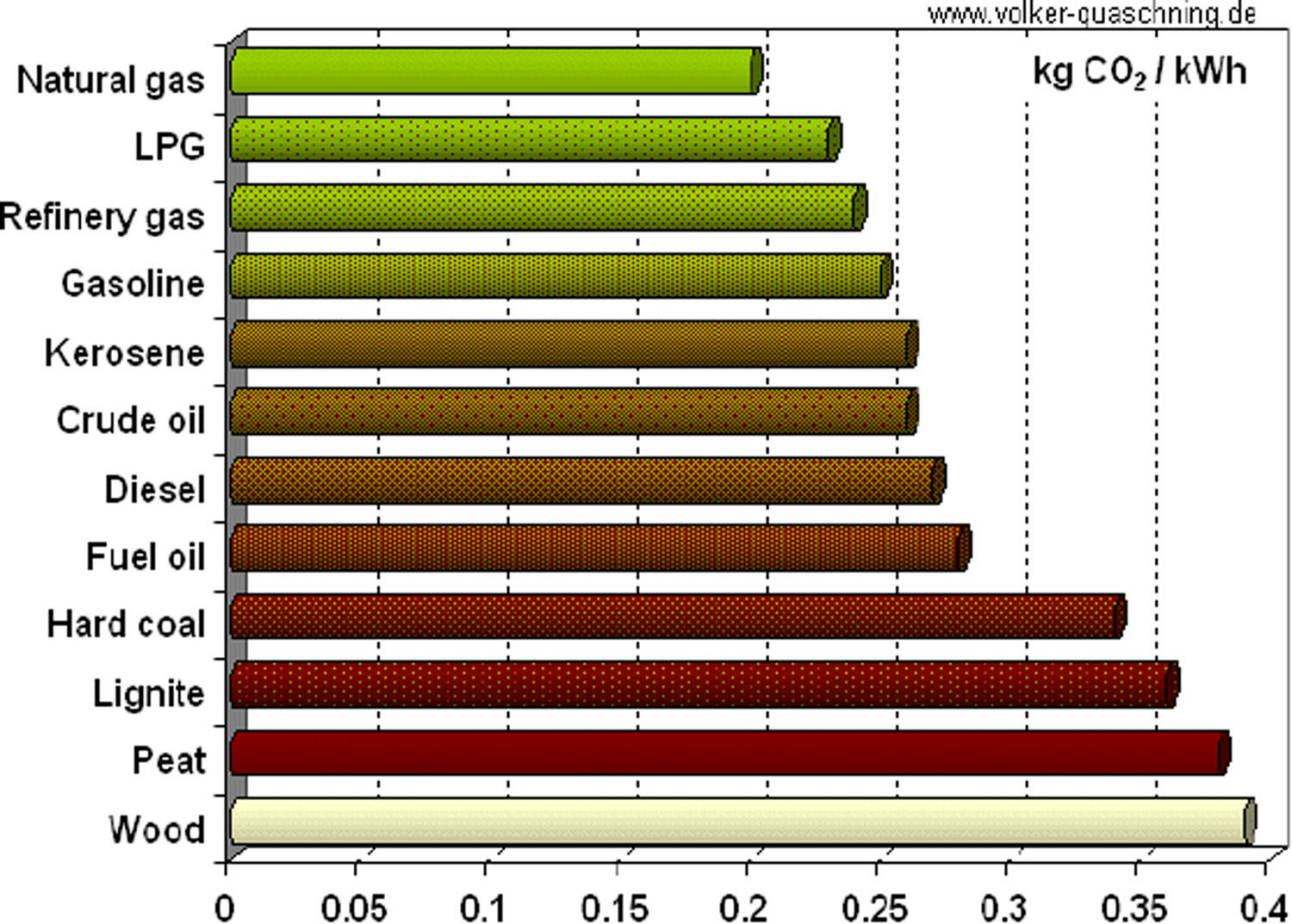
CO_2_ emissions per kWh of electrical energy generated from a range of different carbon sources. Note that, employing these fuels for electricity generation, carbon dioxide emissions increase with the reciprocal of the power plant efficiency. For example, if a power station with an efficiency of 34 % burns coal (58 % for gas), it emits 1.0 kg carbon dioxide during the generation of one kWh of electricity. Reproduced with permission, http://www.volker-quaschning.de/datserv/CO2-spez/index_e.php

*Short-to-medium term (to 2050)* From the perspective of CO_2_ emissions, the promotion of greater penetration of natural gas as the preferred choice as an energy source should be actively pursued. This carbon-based resource may be considered as clean burning (Fig. 1) and as the most appropriate transitional fossil fuel to a low-carbon energy future subject to the qualified assurance that the supply of this gas is substantially leak-free (Alvarez and Paranhos 2012). This does not preclude the need for carbon capture which is imperative and, in parallel with this, short-to-medium term sequestration of CO_2_ emissions will also need to be established.

*Medium-to-long term (2050 and beyond)* To ultimately eliminate carbon emissions while still retaining access to energy dense carbon fuels for utilization in, for example, the commercial transport sector (road freight, shipping, and air), CO_2_ will require capture and re-use. Synthetic fuel production from the captured CO_2_ supported by fuels from bio-resources would see sequestration gradually phased out in the latter half of the century.

## Natural gas resources

**Fig. 2:**
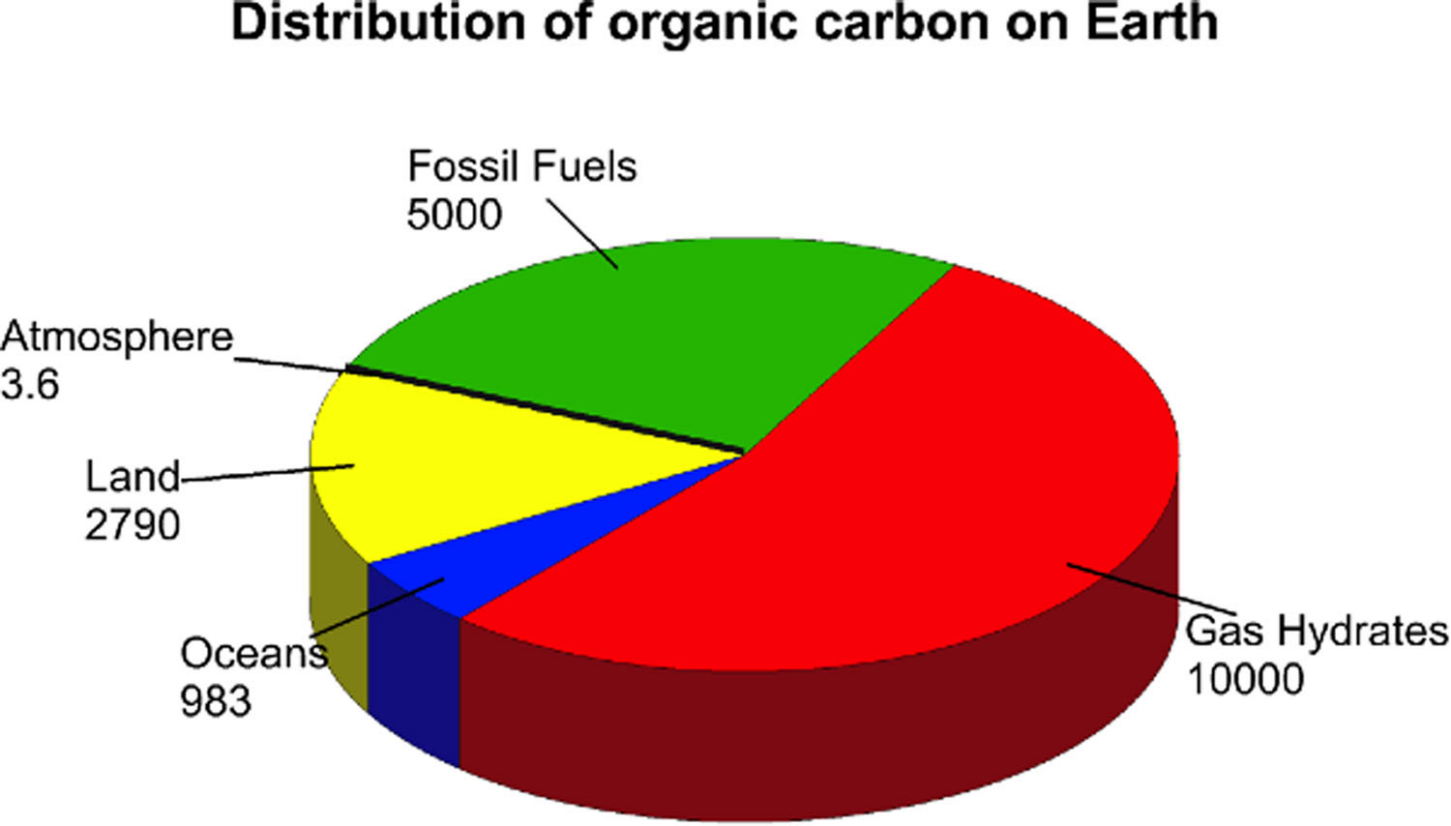
Terrestrial organic carbon distribution in gigatons. Land: soil, biota, peat, detritus; Oceans: dissolved organics, biota; Atmosphere: primarily methane; Fossil fuels: recoverable and non-recoverable coal, oil, and natural gas (carbon in rocks and sediments is excluded and 10^4^ Gt methane in hydrates is equivalent to 14 200 Tm^3^ at STP or 532 000 EJ). Courtesy of the National Oceanic and Atmospheric Administration (NOAA), http://oceanexplorer.noaa.gov/explorations/deepeast01/background/fire/media/carb_dist.html

The cumulative sum of all sources of natural gas across the globe far outweighs existing resources in oil and coal (Fig. 2) and natural gas sources are currently being promoted as key to a new “revolution” in energy availability. Reserves of natural gas, primarily methane, are defined as follows: (i) “Conventional” natural gas: normally sourced in oil fields, isolated gas fields, biogas from contemporary natural decay; (ii) “Unconventional” gas: which exists as coal bed methane, tight gas, and shale gas; (iii) methane hydrates: methane trapped in ice and normally found at depths in the ocean and in polar regions.

Proven reserves of natural gas (IEA 2013), primarily conventional gas with a growing share of unconventional gas in the US and Canada, amount to 187 Tm^3^ (trillion cubic meters) or 6960 EJ (exajoules), while technically recoverable resources including reserves are reported to come to 468 Tm^3^ or 17420 EJ (conventional gas) and 343 Tm^3^ or 12760 EJ (unconventional gas), respectively.^2^ More strikingly, the magnitude of the gas resource in methane hydrates, while uncertain, is considered to be greater than 10 000–20 000 Tm^3^ (the pie chart in Fig. 2 provides a measure of the relative extent of gas hydrate availability compared with other fossil fuels) although recoverability has yet to be assessed. Based on these estimates, with reference to current annual global energy requirements of 470 EJ of which approximately 80 % (375 EJ) corresponds to carbon-based sources, the anticipated lifetime of the combined conventional and unconventional recoverable resources of natural gas, if used in isolation to provide this heat and power, would be ~78 years.

Furthermore, if just 10 % of the resource provided by methane hydrate could be mined and employed under the same conditions this could add as much as a further 150 years to the lifetime of natural gas alone. Utilization of these resources over this period will give rise to a long-term global warming far above present IPCC (2014) projections and more when the emissions associated with the mining of this resource are taken into consideration. A doubling of the total energy demand by 2050 to 940 EJ with 50 % (470 EJ) of this projected heat and power coming from carbon fuels as suggested earlier, would increase the annual release of CO_2_ from approximately 21 Gt CO_2_ per year (if natural gas alone was employed) to 26 Gt per year. The use of other sources of fossil or carbon-based fuels such as oil and particularly coal would only exacerbate this (at this time global annual emissions are at a level of 33.9 Gt).

Clearly, global emissions reduction will require very substantial efforts in improved use of energy (energy efficiency) and the replacement of carbon fuels by non-carbon sources such as nuclear and non-fossil energy. However, in view of recent developments in the area of shale gas and gas hydrates and their projected relevance to the energy landscape in 2050 as implied in the comments above, there are significant economic drivers in place to continue with fossil energy usage which are in turn counterbalanced by a gathering momentum in social and environmental policy.

While conventional gas resources are still being discovered with significant potential in this field in East Africa, the Caspian Sea, Iraq, and the Eastern Mediterranean, in the next section, future prospects for shale gas and gas hydrates will be briefly discussed since these resources could quite probably dominate global carbon energy usage in the latter half of the twenty-first century.

### Shale gas

**Fig. 3:**
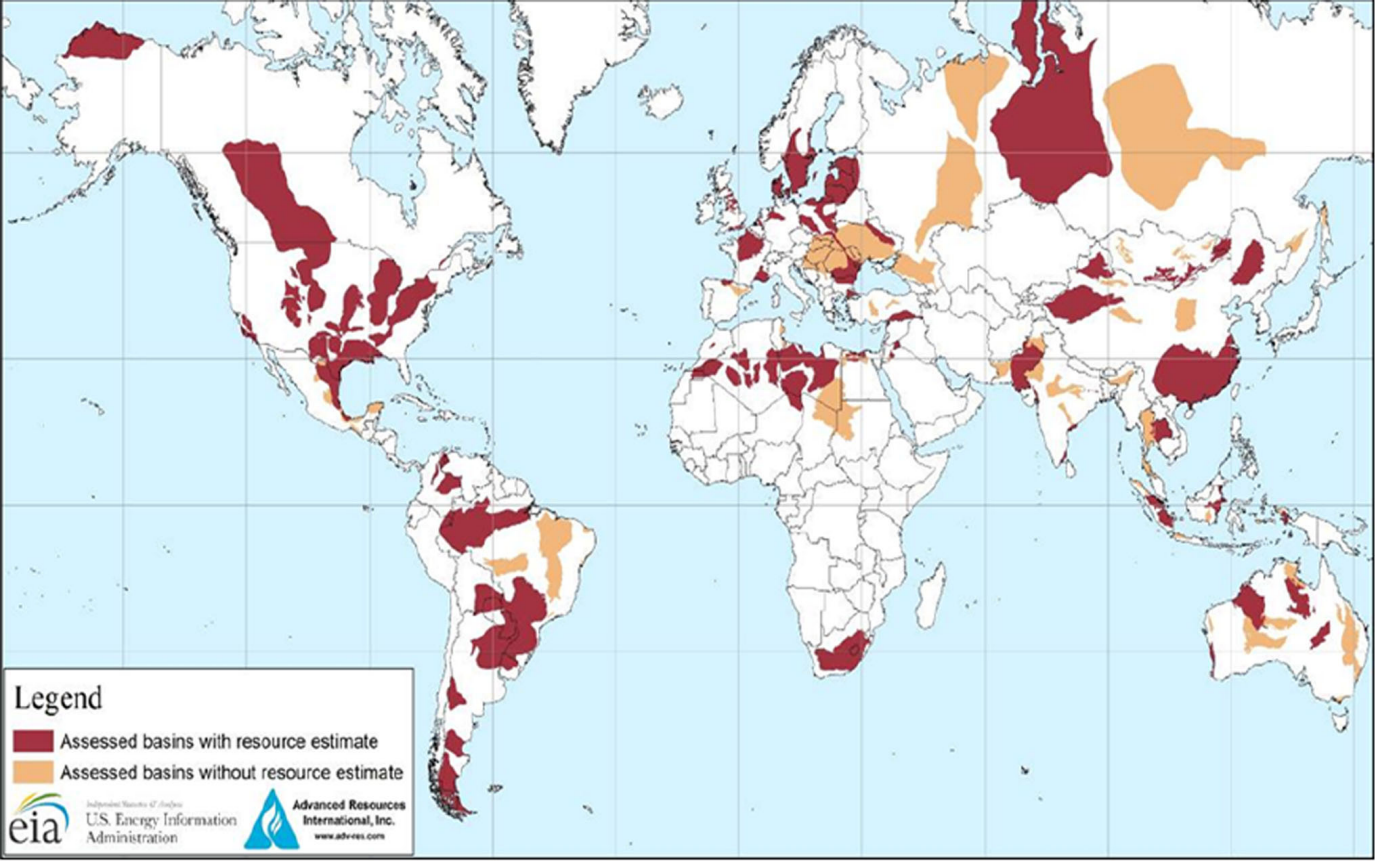
Assessed shale oil and gas basins in various locations across the globe as of June 2013. The *shaded areas* include both shale oil and shale gas. Courtesy of the EIA, http://www.eia.gov/todayinenergy/detail.cfm

Shale gas is natural gas trapped in the cavities of subterranean shale rock which typically stratifies the Earth’s surface over areas of up to hundreds of square kilometers and at depths of the order of a few kilometers. Figure 3 indicates the possible extent of ‘recoverable’ resources as mapped in a recent publication by the Energy Information Administration (EIA). Additional detail relevant to the European sector is provided in a recent document from the European Academies Science Advisory Council (EASAC 2014), wherein questions concerning levels and distribution of shale gas as a resource are discussed at greater length. To access the shale gas, the technology employed is hydraulic fracturing or ‘fracking.’ This technology was originally introduced in 1947 to enhance production of fossil resources (both oil and gas) and with recent significant improvements in the technology subject to social and environmental debate (Howarth et al. 2011; Smith 2012; Weber and Clavin 2012; O’Sullivan and Paltsev 2012; Lutz et al. 2013) fracking is now proving economical on a much broader scale.

While shale gas production is progressing at a significant pace in the U.S. and Canada, in Europe, there has been a decline in both demand and supply of natural gas (van Hittersum 2012). In a report by Altmann and Zittel (2013), a survey of 200 scientific and industrial experts has concluded that for shale gas to see an impetus for large-scale production in the EU, the wholesale price of gas should reach 40–50 €/MWh (the current European price is approximately half this). In a related commentary (Buchan 2013), it has been noted that tension between the EU’s policies on energy and climate change is leading to dissension regarding the development of shale gas resources in Europe. This may be viewed as a boon by those supporting low-carbon technologies but as possibly detrimental to the economic development of the European area by governments and business. In a recent document (EASAC 2014), the direct relevance of three issues to the EU has been acknowledged and addressed: population density, methane leakage, and (local) public acceptance. With regard to population density, recent developments in fracking technology have enabled the implementation of shale gas extraction in areas of high population density and can also infer lower costs and infrastructural demand associated with the shorter delivery distances in such cases. Water quality improvements can also be ensured with stringent application of water management through waste treatment, recycling, and monitoring of groundwater composition. Methane leakage is, however, a serious concern and strict monitoring and implementation of best practices are considered to be a mandatory requirement, particularly with regard to bore well design and construction and the capture of methane and other gases released during the extraction process. To a significant extent, public acceptance of the technology has been dogged by an apparent lack of transparency by the companies developing and operating shale gas extraction sites. For example, although commercial sensitivities may exist with regard to disclosure of the full list of chemical additives employed in the aqueous fracking medium, a level of openness needs to be agreed upon by operatives to encourage confidence in this technology. Developers need to maintain communication with the public and encourage local community involvement in the decision-making process. The EASAC Expert Group conclude that with the experience of shale gas extraction outside the EU and current improvements in fracking technology, exploratory drilling to remove uncertainties relating to extraction viability within the EU should be undertaken. One point worth noting which has had reasonably broad agreement (Buchan 2013) is that shale gas production in Europe will not ensure the same level of energy security as the U.S. currently enjoys although reliance on non-EU natural gas resources could be significantly reduced.

### Methane hydrates

**Fig. 4:**
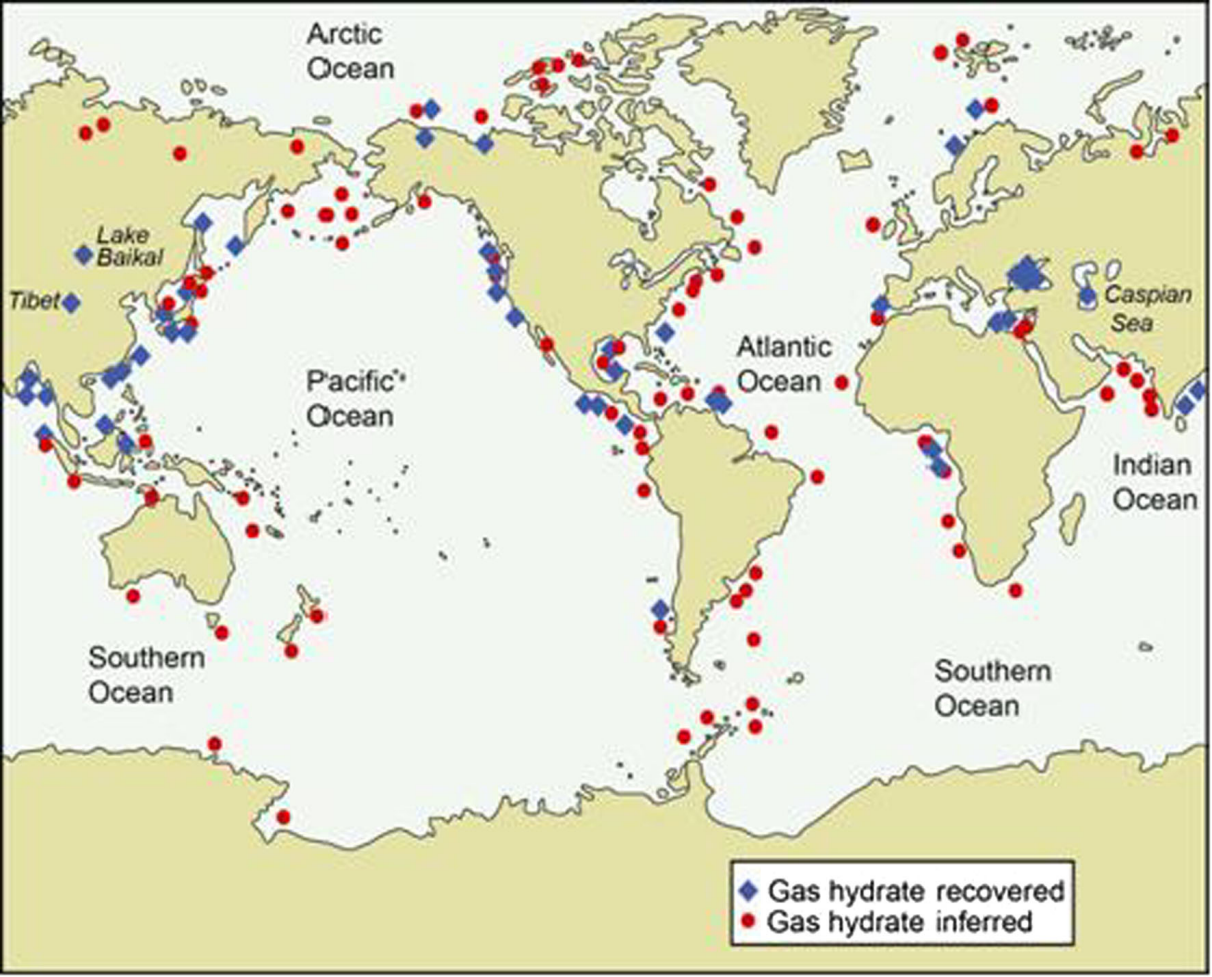
Known and inferred locations of gas hydrate occurrence. Map compiled by, and courtesy of, the US Geological Survey, http://www.usgs.gov

As cited above, methane hydrates far outweigh other sources of natural gas in abundance. These materials are found in the permafrost below 200 m and on the ocean floor at depths >0.5 km and, until very recently, access on a commercial basis to the methane trapped in these ice-like structures (particularly in offshore locations) was believed to be a distant possibility. This changed on March 12th 2013 when the Japan Oil, Gas and Metals National Corporation reached a milestone^3^ and announced successful completion of a test to produce methane gas from offshore hydrate formations off the central Japanese coast for the first time. In view of the vast resource of methane hydrate and its inferred as well as known distribution across the globe (Fig. 4), mining this material in the coming decades might add greatly to environmental problems. A major issue associated with this resource is the risk of uncontrolled release of CH_4_. The concentration of CH_4_ has increased from ~500 ppbv (parts per billion by volume) at the beginning of the industrial era to a current value in excess of 1800 ppbv. Methane itself is 25 times more potent than CO_2_ as a greenhouse gas (Alvarez and Paranhos 2012) and its 1.3 ppmv increase in the last two hundred years or so has approximately 25 % of the effect of CO_2_. The accidental release of even a small portion of this gas during hydrate mining would be catastrophic and tapping this resource would therefore require very stringent regulation and technical oversight in the decades ahead.

### CO_2_ capture

There are two methods available to handle the CO_2_ released during heat and/or power generation using fossil carbon or natural gas:

- storage in geological strata.
- chemical transformation of CO_2_ to products that can be used or are more easy to store.

Both these methods require extensive input of energy, in some cases even more than the energy released in the combustion. The possibility of CO_2_ storage or transformation is therefore strongly dependent on the use of very cheap “waste” energy, such as surplus electrical energy from wind power or technical systems that are based on direct solar energy. Given the current and projected economic pressures for the continued use of carbon-based fuels, and in particular the presumed rapid growth of natural gas usage as implied above, it is imperative that the technology for carbon capture be put in place without delay. In a recent report on carbon capture and sequestration (CCS), the details of this technology have been thoroughly examined (EASAC 2013) and below only a brief summary is provided. This discussion will also focus primarily on the capture component of the technology since it is

(i) the more costly of the two elements of CCS and
(ii) it is felt that in the long term (many decades to centuries), *if* carbon fuels are to be relied on as a primary energy source (the dominant origin of CO_2_ with a modest amount (<5 %) arising from cement production), then the resulting CO_2_ must be re-used or recycled. We will return to the latter in the next section where we outline possible scenarios in which renewable energy is employed in tandem with fossil/CO_2_ reserves.

There are three general technologies which may be applied to capture carbon dioxide from either process streams or from the atmosphere: (i) post-combustion; (ii) pre-combustion; and (iii) oxy-fuel combustion. Each of these methods have one major point in common, they can add a significant cost to the use of carbon fuels in power generation, heating, and transport systems. The fundamental physical chemistry behind these processes is dependent on technology and cost.

Current capture processes would usually operate at 30–40 % efficiency and therefore approximately three times more power would actually be required than indicated in the ideal estimates based on thermodynamics. The dominant operation in each of the three capture technologies outlined above involves the separation of gas mixtures to produce pure CO_2_ followed by gas compression. The minimum power requirements for these separations can be estimated based on the reversible work (free energy change) required for demixing. For example, given that the current EU electric power demand ~450 GW, then it may be shown that the level of additional electric power that would need to be generated in a post-combustion scenario (technology (i) above) to separate and compress (to 100 bar) all European emissions at source (e.g., from power and heating plant effluents and transport system exhausts) with initial CO_2_ gas phase compositions of 5–15 % would be ~50 GW (ideally) or approximately 150 GW in practice. Direct capture from the atmosphere (where the CO_2_ level has now reached ~400 ppmv) would, even under thermodynamically ideal conditions, more than double this power demand and, under realistic conditions, significant breakthroughs in gas handling technology would be required if atmospheric capture was to be considered as an option (see Goeppert et al. 2012, for an interesting overview of techniques for CO_2_ ‘air capture’).

While each of these technologies has their own technical advantages/disadvantages there is significant room for improvement to reduce costs and improve on process efficiencies. For example, breakthroughs will be required in the management and matching of peak and off-peak power loads and the optimization of processes for internal heat recovery through appropriate implementation of process integration (Kemp 2007; Harkin et al. 2010).

Advances in process technology (EASAC 2013) will certainly play a role including: solvent/solution replacement with, for example, ionic liquids in post-combustion capture; novel membrane applications such as advanced hydrogen transport membranes for use in pre-combustion systems and oxygen transport membranes in oxy-fuel combustion separations, with new applications (e.g., adsorption processes) for small-to-medium sized distributed emitters (for use, for example, during location-independent capture from the atmosphere [Goeppert et al. 2012]). New carbon capture technologies are also under development which should play a significant role in driving effective carbon capture power demand much closer to the ideal limits. For example, the Advanced Research Projects Agency-Energy (ARPA-E) Innovative Materials and Processes for Advanced Carbon Capture Technologies (IMPACCT) program is funding projects on supersonic inertial systems with applications for efficient capture of CO_2_ from low concentration gases, novel liquid carbonate/synthetic catalytic processes, and new developments in cryogenic processing with CO_2_ condensation from flue gases (Kramer 2013).

### Utilization of CO_2_

In addition to promoting developments in renewable energy technologies as well as nuclear fusion and fission, in the decades ahead recycle and utilization of the CO_2_ captured from carbon-based heat and power systems (i.e., CCR or CCU rather than CCS) should be given serious consideration if low or near-zero carbon emissions are our ultimate goal. The primary motivation for this is that currently geological storage is considered to be the only viable option for storing CO_2_. The concept of mineral carbonation has significant potential and may provide an effective alternative but research, particularly on associated kinetic processes and, ultimately, environmental impact, is still at an early stage (EASAC 2013). In a European context, the *estimated* capacity of geological storage sites is 117 Gt^4^; however at this time the EU27 generates 3.42 Gt CO_2_ per annum with approximately half of this from large point sources. Therefore, even if full capture was in operation and these sequestration sites were fully accessible now, the utility of subterranean aquifers and depleted gas and oil wells for storing CO_2_ within the EU, even for large point sources, would cease within 60 years and possibly within 30 years if all emissions could be captured.

Furthermore, much of the analysis conducted to date with regard to CCS has been focused on large point sources of CO_2_ which represent approximately half of all CO_2_ emissions, the remainder coming from distributed stationary sources (small-to-medium sized industries, commercial businesses, and dwellings) and mobile sources (road, sea, and air transport). Efforts to improve building energy efficiency and the expansion of electrification to urban transport (cars as well as buses and rail) will focus power demand more on large stationary point sources. However, there will always be a need for high-energy density fuels for road freight, shipping, and air transport which together represent as much as 20 % of global power demand (~3 TW or 100 EJ per annum today and 6 TW in 2050). If near-zero CO_2_ emissions are the ultimate target these distributed mobile sources would require either direct capture from the atmosphere or on-board capture of CO_2_ which, while not impossible, would be technically very challenging. As may be directly inferred from EASAC (2013) carbon capture from the atmosphere should not be dismissed as a possibility when emission sources are widely distributed mobile or small-scale emitters. While the costs of such a process could be high (depending on possible breakthroughs within this field in the coming decades), the advantages that atmospheric capture can offer are that it is not location specific and it does not depend on the specific emission source. On-board capture, however, would depend very much on the emitter. In this regard, it is very unlikely that on-board capture could ever be implemented for air transport. Road freight or shipping are possible candidates for incorporation of small-to-medium scale capture technologies (e.g., adsorption processes, membranes) with interim on-board storage; however costs could be prohibitive (depending on advances within this area) and there is the additional minimum payload of a factor of three for stored CO_2_ relative to fuel.

In the long term (2050 and beyond), to satisfy commercial transport needs in particular, full and continued recycle of captured CO_2_ will be required with fuel production using ‘cheap,’ off-peak renewable energy with little or no need for fossil carbon as a fuel resource. It is important to acknowledge that the economics of such processes must be weighed against the environmental and social benefits which would result since the energy requirements for CO_2_ fixation will generally be significantly higher (by up to a factor of 3 or more depending on process efficiencies) than the magnitude of the stored chemical energy which can be extracted at a later time.

While a number of possible CO_2_ utilization approaches have been discussed in the recent EASAC report (2013), the remainder of this section is focused on possible options that may be developed in the coming decades to employ CO_2_ in chemical synthesis to produce syngas and hence synthetic high-energy density fuels. One possible approach which has a moderate potential for success is liquid-phase water splitting via electrolysis (using off-peak renewable electrical energy) or photo-electrochemical cells (via direct photocatalytic reactions) to produce H^+^, e^−^, and O_2_ followed by condensed phase CO_2_ reduction (see, for example, Schulz et al. 2012; Das and Wan Daud 2014) to produce low molecular weight chemicals.

Another methodology which should see significant breakthroughs in the longer term involves processes which are more closely biomimetic (see, for example, English et al. 2014) and are discussed at greater length in Aro (2016). While these processes are molecularly complex by nature and offer an array of possibilities for breakthroughs in carbon fixation, they suffer a number of drawbacks, the most immediate of which is the solubility of CO_2_ in the dense phases and liquid media (normally aqueous) which are usually involved. Furthermore, there is also the complication of downstream processing of the resulting complex liquid mixture. The methods are, however, conceptually attractive as they mimic photosynthesis and operate at ambient temperatures.

A technology, which would most likely have a greater potential for success in the medium term, would involve gas phase heterogeneous synthesis using renewable energy to produce syngas (CO + H_2_) which may then be converted to fuels and/or chemical feed stocks via the Fischer–Tropsch process. One such approach would involve a two-step process using non-stoichiometric metal oxides (Chueh et al. 2010; Siegel et al. 2013; McDaniel et al. 2013) and concentrated solar power (CSP), for example:

$$ {\text{CO}}_{ 2} + 1/\delta {\text{MO}}_{ 2- \delta } \to {\text{CO + 1}}/\delta {\text{MO}}_{ 2} \quad {\text{Low temperature}}\; ( 8 0 0\;^\circ {\text{C)}} $$

$$ {\text{H}}_{ 2} {\text{O + 1/}}\delta {\text{MO}}_{ 2- \delta } \to {\text{H}}_{ 2} + 1/\delta {\text{MO}}_{ 2} $$

$$ 2/\delta {\text{MO}}_{ 2} \to {\text{O}}_{ 2} + 2/\delta {\text{MO}}_{ 2- \delta } \quad {\text{High}}\;{\text{temperature}}\;(1500\;^\circ {\text{C}},\;{\text{CSP}}\;{\text{driven}}). $$

However, a range of factors concerning this technology needs careful consideration (Siegel et al. 2013). Notably, for viable operation, the thermo-chemical reactor efficiency (thermal-to-chemical energy) should be greater than 36 % and reactor design and the materials of construction of the reactors will require significant development. Reduction in operating temperature and increased conversion using other oxide materials (e.g., perovskites) would also appear to hold significant promise (McDaniel et al. 2014).

A second approach employs solid oxide electrolysis cells (SOEC) (Graves et al. 2011; Laguna-Bercero 2012; Ebbesen et al. 2012) which can produce syngas by high temperature co-electrolysis of both H_2_O and CO_2_.

$$ 2 {\text{H}}_{ 2} {\text{O}} + {\text{ CO}}_{ 2} \to {\text{CO}} + {\text{2H}}_{ 2} + {3}/ 2 {\text{O}}_{2} \quad \left({800{-}900\;^\circ{\text{C}}} \right). $$

This work evolved from the High Operating-Temperature Steam Electrolysis (HOT ELLY) project (Doenitz et al. 1990) of Dornier in the 1980s which originally investigated water splitting alone for the production of hydrogen. The electrolysis aspect was neglected in the 1990s when the focus of attention was directed at solid oxide fuel cell research. SOECs have now attracted renewed interest in the co-electrolysis area and the targets of much of this work relate to improved durability of the cell electrodes and the cost of the technology.

A third possibility currently under development in FOM-DIFFER^5^ (The Dutch Institute for Fundamental Energy Research) involves plasma splitting of CO_2_. The rate of dissociation of CO_2_ is enhanced in the non-equilibrium plasma medium and, in association with water splitting to produce hydrogen, this may ultimately result in an efficient approach for the production of syngas. This work is still in its early stages.

Finally, a technology which is not new (see for example Edwards and Maitra 1995) but has been gaining much attention over the last decade is dry reforming of CO_2_ and methane:

$$ {\text{CO}}_{ 2} + {\text{CH}}_{4} \to 2 {\text{CO}} + {\text{2H}}_{2} \quad \left({700{-}9 50\;^\circ{\text{C}}} \right). $$

The methane for this application should preferably be from contemporary biogenic sources. Furthermore, to obtain the required stoichiometric levels of CO and H_2_ for Fischer–Tropsch synthesis, dry reforming would be accompanied by steam reforming of methane. A broad range of applications, which include dry reforming in conjunction with both the steam reforming reaction and oxygenation of methane, have been reviewed recently (Goeppert et al. 2014).

A major advantage of the dry reforming process is that it fixes two greenhouse gases in one reaction and a number of reactor designs have been proposed which can employ electric power through dielectric barrier discharges (Eliasson et al. 2000) or DC plasmas (Yan et al. 2010) to conduct these endothermic reactions at low temperatures. One of the main issues associated with dry reforming of CO_2_ is coke deposition and catalyst development to overcome this and other kinetic limitations (Fan et al. 2009; Havran et al. 2011; Budiman et al. 2012) has been a driver in the development of heterogeneous systems for this reaction as a route toward CO_2_ emissions mitigation.

Before concluding, one last point needs to be considered in order to assess which, if any, of the above options are viable for consideration as a route or a contribution toward resolution of CO_2_ recycle and re-use. To answer this, a full life-cycle analysis (LCA) is required along lines similar to those described by von der Assen et al. (2013). The environmental impact of each step in the chain of production from capture to chemical synthesis and final product purification and utilization must be carefully accounted for before a given process is adopted for CO_2_ re-use.

## Summary

Ultimately, to reduce the level of carbon-related energy usage (or at the very least attain a steady state with recycling), it is considered that carbon will need to be gradually removed from our inventory of energy resources and excess carbon will need to be stored. Gradual reduction in CO_2_ emissions will initially require a transition to natural gas as the fuel of choice in the short term. There are sufficient ‘conventional’ gas reserves coupled with possible access to a moderate level of ‘unconventional’ (primarily shale) resources (subject to assessment of viability and carbon emissions considerations) to satisfy our needs for this purpose. From an environmental perspective, other sources of gas (in particular hydrates) should be avoided if possible. In the long term (2050 and beyond), to support a target of low or near-zero carbon accumulation within the atmosphere while providing energy dense carbon feedstocks for specific purposes, breakthroughs will be required in a number of areas:

- Projected reliance on energy dense fuels for major components of the transport sector will require either direct on-board carbon capture for mobile emitters (high CO_2_ concentration source) or from the atmosphere (low CO_2_ concentration source) and this should be carefully examined and implemented. In the former case, a substantial payload increase arising from capture infrastructure and CO_2_ storage relative to fuel as well as possible insurmountable technical difficulties in certain cases such as aviation may preclude implementation of on-board capture. Atmospheric capture on the other hand, with the major advantage of capture site non-specificity, would appear to be the more attractive.
- With regard to carbon storage itself, in light of the limited storage capacity available geologically, full CO_2_ (or CO) splitting to solid carbon may be necessary although mineral carbonation may provide an alternative to this approach depending on relative energy demand as well as other process requirements in both cases.
- The necessity for CO_2_ re-use to close the carbon cycle can only be achieved if ‘cheap’ or excess electric power (e.g., from wind or solar) during periods of low demand is employed to produce the fuels required for commercial transport and for the production of chemicals for a number of our needs. While bio-related sources and processes will provide some of the chemical feed stocks required, the author believes that this resource will be insufficient in view of competition with food and the demand for other scarce resources (water in particular). Breakthroughs in catalysis and reactor design will be required to establish novel and efficient synthetic routes in view of the large uphill energy barriers which need to be overcome to convert CO_2_ with H_2_ or hydrogen-rich species back into energy dense compounds.

## Footnotes

1 http://www.iea.org.

2 1 Tm^3^ of natural gas at normal pressure = 37.2 EJ = 10.3 PWh.

3 http://www.bbc.co.uk/news/business-21752441.

4 http://www.cgseurope.net.

5 http://www.differ.nl.
